# 重组人纤维连接蛋白诱导的CIK细胞的生物学特性和对肺癌细胞株杀伤活性的体外研究

**DOI:** 10.3779/j.issn.1009-3419.2010.04.01

**Published:** 2010-04-20

**Authors:** 士勇 王, 微丽 杜, 晖 张, 图雅 乌兰, 远 张, 英 何, 云锋 杨, 飒 刘, 哲 张, 佳玲 王

**Affiliations:** 110032 沈阳，中国医科大学附属第四医院生物治疗科 Department of Biotherapy, Fourth Affiliated Hospital of China Medical University, Shenyang 110032, China

**Keywords:** 纤维连接蛋白, 细胞因子诱导的杀伤性细胞, CD8^+^T细胞亚群, Fibronectin, Cytokine induced killer (CIK) cells, CD8^+^T cells subset

## Abstract

**背景与目的:**

CIK细胞是过继免疫治疗的重要手段之一，简化体外培养过程从而提高其增殖率和杀瘤活性仍是目前研究的一个热点课题。本研究观察重组人纤维连接蛋白（recombinant human fibronectin, RN）诱导CIK细胞的生物学特性，建立一种高效、简便的体外CIK细胞扩增方法。

**方法:**

抽取10名健康人外周静脉血各50 mL，用淋巴细胞分离液分离单个核细胞，分别采用RN诱导法和传统方法培养CIK细胞，记录细胞增殖数；用流式细胞术测定免疫细胞表型和分泌IFN-γ、IL-4、穿孔素和颗粒酶B细胞的百分比；用MTT法测定CIK细胞对4种人肺癌细胞株的体外杀伤率。

**结果:**

RN诱导的CIK细胞扩增倍数为传统方法的2.0倍-3.5倍，具有统计学差异（*P* < 0.05）；RN诱导组和传统方法组CD3^+^CD16^+^CD56^+^细胞绝对数分别增加了3 778倍和2 069倍；RN诱导组细胞中CD3^+^CD8^+^细胞比例明显高于传统方法组（*P* < 0.05）；但CD3^+^CD4^+^细胞比例无统计学差异（*P* > 0.05）；对4种肺癌细胞株的体外杀伤活性无统计学差异（*P* > 0.05）。RN诱导的CIK较诱导前：分泌IFN-γ的细胞比例明显增加；分泌IL-4的细胞比例略有降低；释放穿孔素、颗粒酶B的阳性细胞比例较诱导前增加。

**结论:**

RN诱导法是一种高效、简便的体外扩增CIK方法，可以替代传统方法。

## Abstract (en)

**Background and objective:**

The CIK cell is one of the most important means of the adoptive cellular immunotherapy, and it is a hotspot of which to simplify its culture procedure and to promote its inhibition rate. The aim of this study is to observe the biological function of the CIK cells cultivated by the recombinant human fibronectin (RN) and to establish an effective and simple way of cells expansion.

**Methods:**

We separated the mononuclear cells (PBMCs) in 50 mL peripheral blood from 10 healthy persons with density gradient centrifugation in the lymphocyte-separating medium, and the PBMCs were divided into two groups, of which were cultivated by RN-introduced and conventional method separately. Then we estimated the proliferation ability, and analyzed the immunologic type, IFN-γ, IL-4, perforin and granzyme B of them with flow cytometry. Besides that, we tested the inhibition rate of CIKs cells to four kinds of human lung cancer cell lines *in vitro* by MTT assay.

**Results:**

The RN-induced group had a higher proliferation rate that was 2.0-3.5 times of the conventional group, and there was an obvious statistical difference between the two (*P* < 0.05). The proliferation rates of CD3^+^CD16^+^CD56^+^T cells in each group were 3 778 and 2 068 times of the initial number, respectively. There was also a higher percentage of CD3^+^CD8^+^ T cells in RN-induced group (*P* < 0.05), while the percentage of CD3^+^CD4^+^T cells had no significant statistical difference (*P* > 0.05). We found a similar inhibition rate of the CIK cells to all this human lung cancer cell lines (*P* > 0.05). The cells which secreted IFN–γ increased, while the cells which secreted IL-4 did not. The cells which secreted granzyme B and perforin were positive.

**Conclusion:**

It is an effective and simple way to cultivate the CIK cells with RN, which should be adopted.

近年来，细胞因子诱导的杀伤细胞（cytokine induced killer cells, CIK）因其增殖速度快、溶瘤活性高、溶瘤谱广及对多重耐药肿瘤细胞敏感等优点得到广泛应用^[1-4]^。传统培养CIK细胞方法需要较高的基础细胞数，需用血细胞分离机分离外周血、提取单个核细胞，繁琐耗时，污染机会大，特别不适宜异体供血。如何简化培养过程，提高CIK细胞在体外增殖效率和细胞毒活性是基础研究的一个热点问题。重组人纤维连接蛋白（recombinant human fibronectin, RN）参与淋巴细胞的附着、伸展、分化和增殖^[5]^。本研究初步探讨RN诱导法与传统培养方法在促进CIK细胞体外增殖能力、提高主要效应细胞含量、增强细胞毒活性等方面的优缺点，以期建立更加适合临床应用的CIK细胞的培养方法。

## 1 材料与方法

### 1.1 人肺癌细胞系及培养

肺癌细胞系A549（腺癌）、SPC-A1（腺癌）、CH27（鳞癌）和H460（大细胞癌）购于中国科学院上海细胞库，常规细胞培养，选对数生长期的细胞用于实验。

### 1.2 药物、试剂及主要仪器

抗人CD3单克隆抗体购自美国BD Pharmigen公司；rhIFN-γ购自美国Pepro Techinc公司；rhIL-1购自美国Biosource公司；重组人IL-2为山东泉港药业有限公司产品；RN、无血清培养液KBM551、RPIM-1640培养液为日本宝生物公司产品；佛波酯、离子霉素、莫能霉素、破膜剂、溶血素、三色试剂盒CD8-FITC/CD4-PE/CD3-PC5及CD3-FITC/CD56-PE/CD16-PC5、细胞内细胞因子（IFN-γ、IL-4）测定试剂盒、FITC-抗穿孔素、PE-抗颗粒酶、APC-抗CD8荧光抗体均为美国BD公司产品。流式细胞仪为美国BD公司生产，型号FC 500 MPL。

### 1.3 CIK细胞的体外培养、扩增及存活率测定

抽取10例健康人（男、女各5例，年龄37岁-57岁）外周静脉血每人50 mL，用淋巴细胞分离液提取外周血单个核细胞（PBMCs），每份血样的PBMCs均分为2份，采用2种方法诱导培养，即RN诱导组和传统方法组。RN诱导组采用新培养方法：即用RN（25 μg/mL）和抗CD3 mAb（5 μg/mL）提前24 h包被培养瓶，以后培养体系中只加入IL-2（1 000 U/mL）和KBM551无血清培养基。传统方法组：提前24 h用抗CD3 mAb（5 μg/mL）包被培养瓶，培养当天加入重组人IFN-γ（1 000 U/mL）、IL-1α（500 U/mL）、IL-2（1 000 U/mL），以后仅加入IL-2（1 000 U/mL）和KBM551无血清培养基。培养条件是在37 ℃、5%CO_2_细胞培养箱内培养。每3天用台盼蓝染色检测细胞存活率，记录细胞绝对数。

### 1.4 淋巴细胞免疫表型检测

分别取培养第0、7、14、21天样品6 mL，离心后用PBS洗涤2遍，按照BD公司试剂盒说明书染色，采用流式细胞仪测定CD3^+^、CD3^+^CD4^+^、CD3^+^CD8^+^、CD3^+^CD16^+^CD56^+^细胞的百分比。

### 1.5 CIK细胞体外杀瘤率测定

采用MTT法，选对数生长期的上述4种肺癌细胞株，调整细胞浓度为8×10^4^/mL，于96孔板中每孔加入100 μL，37 ℃、5%CO_2_培养箱中培养4 h。取培养第15天的CIK细胞为效应细胞，按效:靶比20:1和40:1加入CIK细胞，设效应细胞杀伤组、效应细胞对照组、肿瘤细胞对照组，每组设6个复孔。共同培养48 h后加入2.5 mg/mL MTT溶液40 μL后再培养4 h。离心弃上清，加入150 μL DMSO溶液震荡混匀，酶标仪选择570 nm波长，测定各孔光密度值（optical density, OD），计算杀瘤率，计算公式为：杀瘤率（%）=1-[（杀伤组OD值-效应细胞对照组OD值）/肿瘤细胞对照组OD值]×100%。

### 1.6 细胞内细胞因子、穿孔素和颗粒酶检测

取培养第15天的CIK细胞悬液0.5 mL，调整细胞数为1×10^6^/mL，经佛波酯、离子霉素激活、莫能霉素阻断4 h，加入APC荧光标记的抗CD3 mAb，室温、避光15 min标记细胞膜表面分子；而后加入破膜剂和抗IFN-γ、IL-4、穿孔素、颗粒酶B等荧光标记的单克隆抗体，室温、避光30 min后，用PBS洗去多余的抗体，用流式细胞仪测定CD3^+^细胞中表达IFN-γ、IL-4、穿孔素、颗粒酶B的细胞的阳性率。

### 1.7 统计学处理

所有数据使用SPSS 11.0软件进行统计学分析，试验结果用Mean±SD表示，双侧*t*检验比较两组均数的差异，*P* < 0.05为差异有统计学意义。

## 2 结果

### 2.1 两种方法诱导CIK细胞的增殖活性与存活率

RN诱导组和传统方法组的CIK细胞均表现出非常高的增殖活性（见图 1），从起始细胞数的（2.35±1.13）×10^7^，至第22天达高峰，CIK细胞数分别达到（750.00±109.23）×10^7^和（343.00±188.70）×10^7^；而且，从培养第7天-第28天，RN法高出传统法2倍-3.5倍，有统计学差异（*P* < 0.05）。检测RN诱导组和传统方法组的CIK细胞存活率，第15天分别为（97.71±1.07）%和（95.48±2.01）%，第21天分别为（92.23±2.28）%和（90.85±5.28）%，存活率都在90%以上。

**1:**
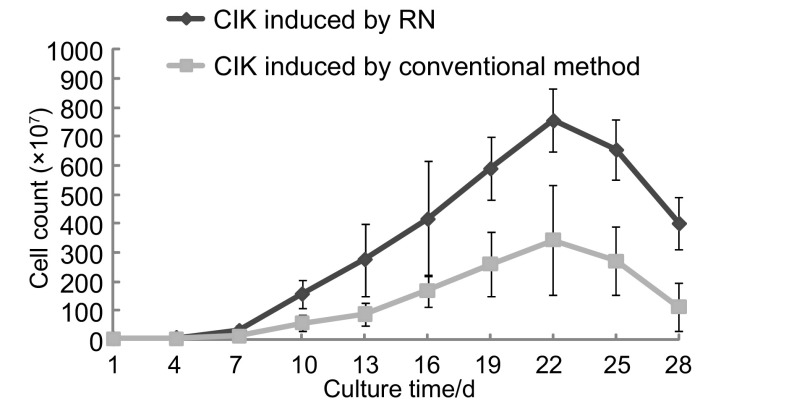
2种培养方法诱导的CIK细胞的增殖能力 Proliferation ability of CIK cells cultured by two methods RN: recombinant human fibronectin.

### 2.2 RN诱导CIK细胞免疫表型的变化

随着体外培养天数的增加，RN诱导组和传统方法组CD3^+^细胞比例均上调（见表 1和图 2，传统方法组流式结果图未给出），至第21天，分别达到（97.08±2.14）%和（97.22±2.25）%，CD3^+^CD4^+^细胞比例分别下降至（15.31±8.49）%和（18.79±6.84）%，CD3^+^CD8^+^细胞比例分别上升到（76.26±8.60）%和（66.79±9.52）%；CD3^+^CD16^+^CD56^+^细胞比率也上升，由初始（1.83±0.212）%分别上升到（26.01±7.68）%和（31.32±7.34）%，且绝对数分别增加了3 778倍和2 069倍。与传统方法组比较，RN组显著增加了CD3^+^CD8^+^细胞的比率，在第14天增幅达高峰，为（77.30±6.72）%，显著高于传统方法组（68.31±8.87）%（*P*=0.02），直到第21天CD3^+^CD8^+^细胞仍然保持较高的比率，分别为（76.26±8.61）%和（66.79±9.52）%，差异具有统计学意义（*P*=0.042）；RN组对CD3^+^CD4^+^细胞比例的影响，与传统方法组相比，第7、14、21天在数量上相似（*P* > 0.05）。另外，RN诱导组的CD3^+^CD16^+^CD56^+^细胞比例低于传统方法组，经统计学处理，第7、14、21天*P*值分别为0.389、0.439、0.153，均大于0.05。而且，由于RN诱导组第7天-第21天细胞总数明显高于传统方法组，故CD3^+^CD16^+^CD56^+^细胞绝对数较高。

**1:** RN诱导法培养的CIK细胞中各亚群比例随培养天数的变化 The ratio changes of the CIK cells subsets cultured by RN

Phenotypes	CIK cultured by RN					CIK cultured by conventional method			
	d1	d7	d14	d21		d1	d7	d14	d21
CD3^+^	73.48±4.95	86.07±2.74	92.54±1.78	97.08±2.14		73.48±4.95	81.31±3.64	91.45±1.78	97.22±2.25
CD3^+^CD4^+^	60.00±8.61	31.86±12.09	24.10±13.39	15.31±8.49		60.00±8.61	34.64±14.35	26.88±14.11	18.79±6.84
CD3^+^CD8^+^	31.02±2.57	65.12±10.86	77.30±6.72	76.26±8.60		31.02±2.57	62.20±13.73	68.31±8.87^*^	66.79±9.52^**^
CD3^+^CD16^+^CD56^+^	1.83±0.21	4.43±2.35	15.16±8.49	26.01±7.68		1.83±0.21	5.33±2.25	18.21±8.73	31.32±7.34
^*^t =2.55, *P*=0.020; ^**^t =2.21, *P*=0.042.									

**2:**
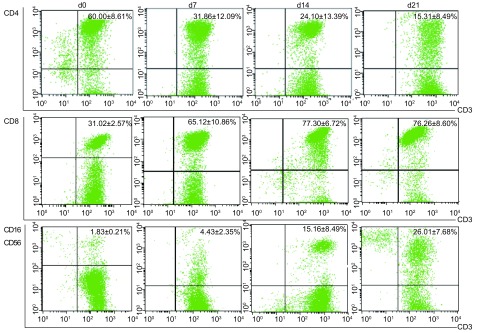
RN诱导法培养的CIK细胞中各亚群比例随培养天数的变化 The ratio changes of the CIK cells subsets cultured by RN

### 2.3 CIK细胞体外杀瘤率的比较

RN诱导组和传统方法组的CIK细胞，对4种肺癌细胞株体外杀瘤率比较，在同一效:靶比水平上无统计学差异（*P* > 0.05）。但将2种培养方法诱导的CIK细胞数量增加时，效:靶比40:1组的杀瘤率均明显高于效靶比20:1组（*P* < 0.05），结果见表 2。

**2:** 两种方法诱导的CIK细胞杀瘤率比较（%） The inhibition rate of CIK cells cultured by two methods (%)

Cancer cell lines	Effect:Target=20:1			Effec:Target=40:1	
	RN method	Conventional method		RN method	Conventional method
A549	57.54±18.37	50.28±10.66		74.28±15.71	74.80±7.69
SPC-A1	58.58±8.52	61.11±8.66		80.65±9.42	82.56±15.48
CH27	32.47±7.51	43.34±7.68		65.43±4.91	61.75±6.02
H460	48.43±8.15	46.51±5.54		71.60±6.49	68.90±5.13

### 2.4 RN组CIK细胞的细胞因子、穿孔素和颗粒酶B的检测

RN诱导的第15天CIK细胞，分泌IFN-γ的细胞比例增加，从诱导前24.89%增加至58.76%；分泌IL-4的细胞比例变化不大，从诱导前2.16%下降到0.53%（图 3）；释放颗粒酶B、穿孔素的细胞也呈阳性（图 4），诱导前为阴性。

**3:**
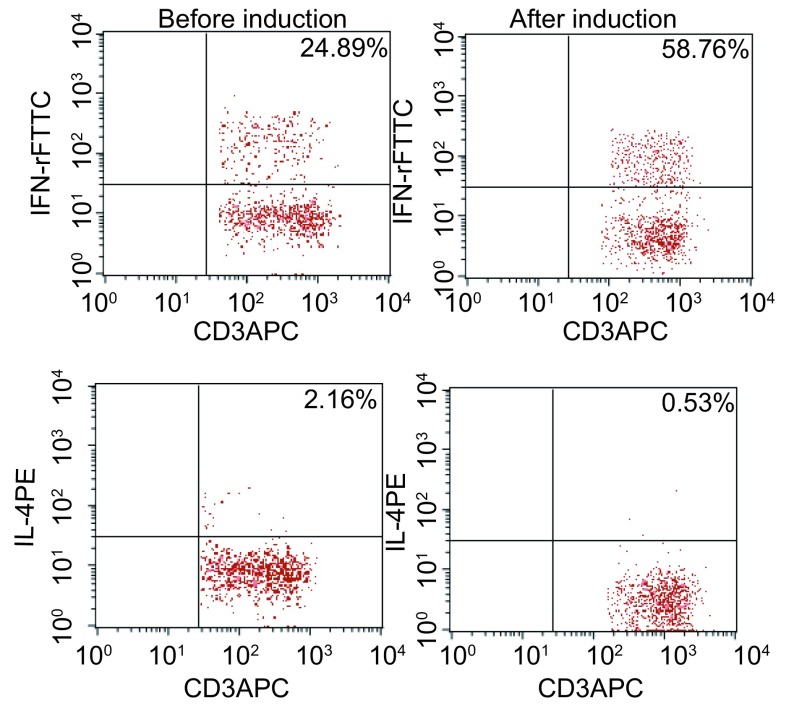
RN诱导前后分泌IFN-γ和IL-4细胞的阳性率 The positive rate of cells secreting IFN-γ and IL-4 in CIK cells cultured by RN

**4:**
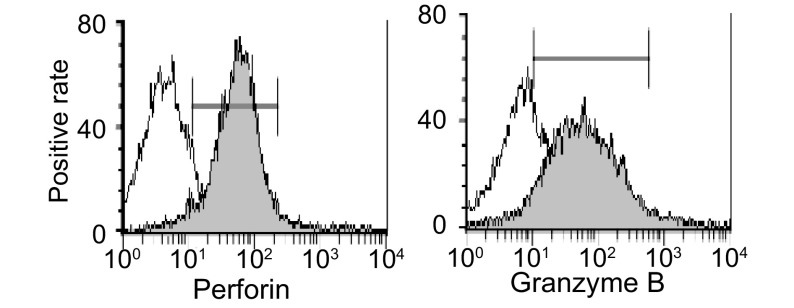
RN诱导后的CIK细胞穿孔素、颗粒酶B的阳性率 The positive rates of cells secreting perforin and granzyme B in CIK cells cultured by RN

## 3 讨论

RN是重组人纤维连接蛋白片段，包括细胞结合域、肝磷脂结合域和CS1位点三个功能区域，由574个氨基酸组成，分子量为63 kDa。其生理活性为参与淋巴细胞的附着、伸展、分化和增殖。RN和抗CD3抗体可分别与T淋巴细胞上的VLA和TCR结合，两者共同作用激活酪氨酸激酶pp125FAK，然后通过Ras途径刺激T细胞的增殖和分化^[5]^。本实验中新引入了RN，并首次系统地进行了生物学特性的研究，获得了比传统培养方法更高的促淋巴细胞增殖效果，从细胞数量和质量上保证了CIK细胞回输治疗。新培养方法采用抽取50 mL外周静脉血的方式代替血细胞分离机，减少了对供体免疫功能的影响和感染机会；以RN替代了IFN-γ和IL-1α，降低了总成本。

本研究结果显示，健康人外周血淋巴细胞经特定的细胞因子诱导及体外扩增培养后，CD3^+^、CD3^+^CD8^+^、CD3^+^CD56^+^CD16^+^细胞比例较培养前明显增加，CD3^+^CD4^+^及CD3^+^CD4^+^与CD3^+^CD8^+^的比值则明显降低，即杀伤性T细胞比例增加，辅助性T细胞减少。CD8^+^T细胞是一群异质性的细胞，经过刺激后“原始”CD8^+^T细胞能增殖、分化为效应性T细胞或记忆性T细胞。效应性T细胞可通过分泌穿孔素、颗粒酶等物质直接杀伤靶细胞，但其增殖能力弱，一旦机体内抗原的浓度减低或消退后效应性CD8^+^T细胞的数量急剧减少。记忆性CD8^+^T细胞经初次抗原反应后能存活较长时间，若再次受到相同抗原刺激它能迅速地被活化并分泌大量的细胞因子。有研究^[6-8]^结果说明培养后CD8^+^T细胞是以记忆性细胞而不是以“原始”细胞为主，使得培养后的细胞具有一定的增殖潜能，更有利于机体的抗肿瘤反应。表面标记为CD3^+^CD56^+^CD16^+^的细胞被认为是CIK细胞的主要效应细胞，在体外扩增过程中可扩增至2 000倍以上。以往研究^[9]^表明CD3^+^CD56^+^CD16^+^细胞80%以上来源于CD8^+^杀伤性T细胞，理论上讲伴随CD3^+^CD8^+^细胞的增多，在回输至患者体内后转化为CIK细胞的比例也会增加。

体外细胞毒性试验证实CIK细胞对多种肺癌细胞株都具有较强的杀伤力，其杀伤活性随着效应细胞比例的增大而增加。RN诱导的CIK细胞与传统方法获得的CIK细胞对同一种瘤细胞的杀伤活性相当。

本研究初步探讨了CIK细胞的杀瘤机理。健康人PBMCs经体外RN诱导后的CIK细胞遇到抗原刺激很快被激活并分化为具有细胞毒性的效应细胞，分泌Th1型细胞因子IFN-γ，高表达TNF-α、Perforin等细胞坏死相关因子，几乎不分泌Th2型细胞因子IL-4，因而在免疫应答中倾向于Th1优势。

RN诱导的CIK细胞与传统培养方法相比，具有更强的体外增殖能力，增加了杀伤性T淋巴细胞的比例，并且可能带来提高远期杀瘤活性的优势。其体外杀瘤活性与传统诱导方法作用相当。分泌杀伤性细胞因子、释放颗粒酶、穿孔素的水平较激活前明显增多。此外新培养方法所需血量少，对供体免疫功能影响小，使用无血清培养基减少了外源感染的机会；在我们的临床应用研究中，RN诱导的CIK细胞单独或与化疗等其它治疗方法联合应用，表现出明显的优势，抽血时间方便，不受病人的状态和治疗的影响，增加病人的抵抗力、体力，延长了病人的生存期^[10]^，值得推广应用。
